# A Single Drop in the Eye – Effects on the Whole Body?

**DOI:** 10.2174/1874364101711010305

**Published:** 2017-10-31

**Authors:** Anu Vaajanen, Heikki Vapaatalo

**Affiliations:** 1 Department of Ophthalmology, Tampere University Hospital, P.O. Box 2000, 33521 Tampere, Finland; 2 SILK, Department of Ophthalmology, School of Medicine, University of Tampere, 33014, Tampere, Finland; 3 Medical Faculty, Pharmacology, University of Helsinki, P.O. Box 63, 00014, Helsinki, Finland

**Keywords:** Single drop, Ophthalmic drugs, Eye, Body, Kinetics, Ocular drugs

## Abstract

**Introduction:**

Although the local adverse effects of ophthalmic drugs, including allergic reactions, are well recognized, less is known about the systemic side- effects of eye drops, especially during pregnancy, breast-feeding and early childhood. Ophthalmologists should also be aware of unusual, in some cases even life-threatening, effects of commonly used eye drops.

**Conclusion:**

This brief review outlines the routes of systemic absorption and the kinetics of active components present in eye drops, and identifies the clinically relevant systemic adverse effects.

## 1 INTRODUCTION

Local therapy, as administered in dermatology, in lung, ear and nose diseases as well as in ophthalmology, is assumed to exert its effects in the organ treated, but can in fact also evoke systemic, mainly harmful effects if the drug is absorbed into the circulation. It is nearly fifty years ago since the first case reports of acute elevation of blood pressure in pediatric patients after cyclopentolate- [1] or phenylephrine- [2, 3] containing eye drops were published. The increase in blood pressure seen in low birth weight infants was apparently attributable to large doses/high drug concentrations in the eye drops used. The mucosal epithelium in the mucous membranes of the conjunctiva and nose is readily permeable to drugs, allowing them to gain access to the systemic circulation without being subjected to first- pass metabolism in the liver. Aged patients are another at- risk population; timolol for example has exerted systemic adverse effects causing bradycardia, a fall in blood pressure and bronchoconstriction [4, 5]. Many of the adverse effects experienced by the elderly are associated with polypharmacy and the simultaneous treatment of different diseases. Pregnant women or nursing mothers comprise another group of at- risk patients with respect to ophthalmic drugs. Recently, more has been learned regarding the systemic effects of ophthalmic drugs and it is now appreciated that they may not merely be distressing but in some cases even life threatening. We herein provide a short overview of what is known about drug absorption and transport from the eye to the circulation and subsequently to various organs in the body.

### 1.1 Kinetics of Drugs in the Eye and Their Transport from the Eye

The pharmacokinetic aspects of locally administered ophthalmic drugs display many special characteristics in comparison to other routes of administration. This topic has in fact been thoroughly discussed in a recent review [6]. The size of the drop as well as the vehicle used and the lipophilicity/hydrophilicity of the active compound, *i.e.* its physicochemical properties, as well as its concentration in the preparation, are the main determining factors. Lipophilic drugs are absorbed well through the cornea, whereas both lipophilic and hydrophilic compounds are well able to penetrate through the conjunctiva and sclera. The vitreous humor can transport drugs via the blood-retinal barrier, lipophilic compounds being more readily transported. There is also a route out of the vitreous humor via the anterior chamber, through which both hydrophilic and lipophilic drugs can be excreted [6, 7]. When eye drops are dispensed onto the surface of the eye, they remain there for only a short time even when ophthalmic ointments are used. If the patient presses the medial eye-nose corner (so-called puncta compression), this can retard the diffusion of the drug into the lacrimal drainage. It has been estimated that the ocular bioavailability of the active compound in eye drops is very poor, a mere 5 -10%. Other factors can contribute to their low bioavailability, namely binding to proteins and metabolizing enzymes in the lacrimal fluid, the thickness of the corneal surface and lacrimal film as well as conjunctival hyperemia [6].

The precise role of the influx transporters, *e.g.* organic cations and peptides, can influence penetration through membranes, depending on the chemical nature of the particular agent in question [8]. Furthermore, the presence of drug- metabolizing CYP enzymes in the ocular tissues can be of importance *e.g.* in the case of timolol [9]. There are also other enzymes, *e.g.* esterases, in the eye; which are important in that they metabolize prodrugs into their active form, as for example dipivefrin to adrenaline, latanoprost to prostaglandin F2alpha [6]. Orally administered drugs, which inhibit various drug- metabolizing CYP enzymes, can retard the metabolism of ophthalmic agents after their absorption into the eye, and on the other hand, can cause systemic adverse interactions after the topical drug has gained access to the circulation [10]. For this reason, patients who are poor metabolizers of CYP2D6 or are receiving drug treatment with potent inhibitors of this enzyme (*e.g.* paroxetine, fluoxetine or verapamil) are at- risk of adverse cardiac effects (*e.g.* bradycardia) when using timolol or betaxolol eye medication [11].

Drugs applied in the “cul-de-sac” encounter many drug- metabolizing enzymes existing in the ocular tissues and fluids. Some metabolism of the drugs can occur already in the ocular tissues. Furthermore, the proportion of the drug which is absorbed into the mucosal membrane of the nose can bypass the first- pass metabolic processes in the liver and reach its target tissues more readily than if given orally. It has been estimated that up to 80% of a drug applied onto the conjunctival sac may diffuse into the systemic circulation via the highly vascularized nasopharyngeal route [6]. Other minor but nonetheless important routes of systemic absorption are the conjunctiva (increased with hyperemic drugs), lacrimal drainage, the skin of the cheek and eyelids, aqueous humor and even the inner parts of the eye.

Intravitreal injection is a special form of ocular application of drugs, often utilized for more recent biological preparations [12]. In this case, these compounds are removed either across the vitreous body in the posterior chamber and then onwards to the aqueous and uveal circulation across the blood – ocular barrier passively or by exploiting active transporter systems [6, 13]. Interestingly, it has been speculated that intravitreal anti-VEGF drugs could cause systemic thromboembolic adverse events [14], and thus are usually clinically contraindicated during the first year after cardiac or brain ischemic events. More evidence will, however, be needed to confirm these suspicions.

In view of the possibility that ophthalmic drugs can exert systemic adverse reactions, we surveyed the Finnish Drug Manual for Physicians (Pharmaca Fennica) to identify the most common systemic adverse effects of ocular drugs; these are summarized in Tables (**1** and **2**).

In the following sections we will present more detailed data on four especially at-risk clinical groups (children, pregnant and nursing women and elderly subjects), as well as some rare clinical problems.

### 1.2 Children

Newborns and infants require special attention by reason of their anatomical differences in comparison to adult eyes. The absorbing membranes are thin and drug penetration can thus be rapid, the tear volume is small, meaning that the drug concentration in the eye is higher than in adults. Young children up to school age need “smaller” drops and/or lower concentrations of ophthalmic preparations [6, 15], but these are unfortunately rarely available. Similarly to the situation with other medicines, few kinetic or therapeutic studies of ophthalmic agents in the case of children have been published; those in the literature often comprise case reports depicting toxicity. Mydriatic drugs, *e.g.* cyclopentolate, which are widely used in the ophthalmological examination of pediatric patients, can trigger to respiratory distress [16], cyclopentolate and phenylephrine can evoke myoclonic seizures [17, 18]. Furthermore, they may cause skin rash, tachycardia, feeding problems, discomfort, apnea, gastric dilatation and ileus. Ozgun and associates 2014 [19] reported a lethal case of diffuse necrotizing enterocolitis after topical application of drops containing 0.5% cyclopentolate and 1.25% phenylephrine. Phenylephrine, on the other hand, can cause hypertension, ECG changes and elevation in cardiac troponin Ic [20]. However, the case report in question seems to be exception, since a systematic review and meta-analysis [21] found no evidence that phenylephrine 2.5% evoked clinically relevant changes in heart rate, and the reported elevation in blood pressure was short lasting. In clinical practice, mydriatic eye drops (tropicamide and phenylephrine 2.5%) are widely dispensed for small infants, for example to investigate premature retinopathy.

Anti-glaucoma drugs are used in the treatment of childhood glaucoma; however, none of the drug concerned has been approved by the regulatory agencies for administration to children, *i.e.* they are being used on an off-label basis. Once- a -day application of timolol gel for some time now has been the first choice in pediatric glaucoma. Second choice is a combination of timolol once a day and a carbonic anhydrase inhibitor (CAI) twice a day. Both medications are effective and well tolerated, though in theory, timolol drops can cause severe bronchoconstriction and CAI may induce metabolic acidosis [22, 23]. Betaxolol solution is a selective beta-1 antagonist, and thus involves a lower risk than timolol of triggering pulmonary side- effects [23]. The safety profile of prostaglandin analogues seems to be excellent also in children, although they are not recommended for use in infants under one year of age due to poor knowledge of their adverse effects. The kinetics and systemic effects of latanoprost have been evaluated in one study using once daily treatment; it was concluded that systemic exposure was more marked in younger children than in adolescents and adults. The drug was rapidly eliminated in all age groups, and no clinically significant adverse effects were reported in any [24]. According to several reports, anti-glaucoma therapy with alpha2-agonists (*e.g.* brimonidine) is contraindicated for children younger than 2 years of age [23, 25-27], there is even one claim that these drugs should not be administered to children younger than 6 years and weighing less than 20 kg [23]. The reason for these recommendations is that alpha2-agonists such as brimonidine have evoked severe central nervous depression, sleepiness, lethargia, hypotonia, hypothermia, bradycardia and apnea in infants [23-25, 28].

Chloramphenicol is one of the most widely used antibiotic ophthalmic preparations for the treatment of bacterial conjunctivitis in infants. Allergic reactions are possible. There are some anecdotal, poorly defined, cases in the older literature of chloramphenicol-induced bone-marrow hypoplasia and blood dyscrasias [29, 30, 31]. It is possible that there had been previous chloramphenicol exposure in the form of oral therapy and the side- effects attributed to the eye drops were thus in fact a secondary immunological reaction. Orally administered chloramphenicol is contraindicated in neonates due to the life- threatening “gray baby syndrome” [32]. Other local ophthalmic antibiotics such as fluoroquinolones are usually taken to be contraindicated for infants under one year of age because of the lack of knowledge of their potential side effects.

Corticosteroid eyedrops are valuable in the treatment of intraocular endogenous inflammation such as juvenile rheumatoid arthritis associated eye diseases and other uveitic conditions. A few reports have been published on significant systemic adverse effects of corticosteroid drops. When corticosteroids are absorbed via the ocular or nasal mucous membranes, they do not undergo first-pass metabolism in the liver. Even locally administered steroids may cause adrenal suppression after prolonged administration, especially in children [33]. There are conflicting opinions on the ophthalmic use of corticosteroids in small children and their effects on growth. Wolthers (2011) utilized a novel method, knemometry; he reported that two weeks´ treatment with the synthetic corticosteroid, fluorometholone, did transiently suppress growth, but this had no impact on height gain during the following year [34].

### 1.3 Pregnant and Nursing Women

There are limited data on this topic as prospective and randomized clinical trials are subject to ethical and legal constraints in this patient population [35]. The smallest dose/concentration and shortest possible exposure time are recommended in these patients. As a general rule, drugs should (if possible) be especially avoided in the first trimester during the time of organogenesis [36]. Razeghinejad and collagues stated that according to the FDA classification, anti-glaucoma drugs should be categorized as class C [37], *i.e.* ”risk for the fetus cannot be ruled out”. The exceptions are brimonidine and nonspecific adrenergic agonists, which have been categorized as class B “no evidence of risk”. The FDA group C also includes tobramycin, ciprofloxacin, phenylephrine, latanoprost and corticosteroids.

In patients with severe high-pressure glaucoma *e.g.* secondary glaucoma due to uveitis, it is essential to administer anti-glaucoma drugs even during pregnancy. In these cases it is important to ensure that the patient presses her nasolacrimal puncta for a couple of minutes after each drop. In addition, laser treatment of the trabecular meshwork is preferable as this obviates the need for drug therapy. The first trimester of pregnancy, *i.e.* the period of organogenesis, should be kept as a drug-free period [38]. Beta-blockers should be avoided due to their hazardous effects on the fetus such as retardation of growth, cardiac conduction disorder, even teratogenic effects.

Beta-blockers are actively excreted into breast milk and can be harmful for the newborn. These infants should be closely monitored for signs of apnea and bradycardia. The excretion of most drugs into the mother´s milk is negligible; timolol seems to be an exception, as it has been detected in milk after a single drop of 0.5% timolol (case reports [39] and [40]). Interestingly, obstetricians widely prescribe oral beta-blockers to control hypertension during pregnancy, apparently without any major problems, but it is known that systemically administered timolol is not able to reduce IOP [38]. The peak concentration of topically administered eye drops in the systemic circulation is reached within 30-120 minutes after application. If possible, the drug administration should be scheduled to avoid the time when the mother is suckling her child. However, there is no consensus as to the use of timolol; Lustgarten and Podos: “timolol should be used with caution by nursing mothers”, Madadi and associates “this dose of timolol is unlikely to cause systemic side- effects in a healthy breastfed infant” [39, 40].

Oral carbonic anhydrase inhibitors (acetazolamide) at high doses have evoked birth defects in animals [41-43], but there is little clinical evidence in support of such an outcome in humans, though a single case of a sacrococcygeal teratoma [44], transient renal tubular acidosis [45] and a single case of suspected congenital malformations [46] have been described. There are no reports of fetal complications following topical use of carbonic anhydrase inhibitors in pregnant women or abnormal reactions in breast-fed infants [37], though these compounds were found to retard the body weight of dog pups.

All prostaglandin analogues have been classified by FDA into category C due to their capability to stimulate uterine smooth muscle contraction, but they are considered a reasonable option during lactation [35]. They are excreted into breast milk in animals, but it is not known whether this is also the case in humans [37].

Anticholinergics are also classified as category C since in experimental animals they have been shown to be teratogenic and to cause adverse effects [37, 47].

Sympathomimetics such as alpha-2-agonists can surprisingly be used during pregnancy (category B) but are contraindicated during nursing as possible causing severe nervous system depression in breast- fed infants.

In conclusion, none of the anti-glaucoma drugs has been shown to be teratogenic in humans but on the other hand, none can be said to be risk-free. In some cases, when necessary, even glaucoma surgery can be performed with caution, preferably after the first trimester, although, anesthetics, sedative agents and antimetabolites are of course also potentially risky for the fetus [46].

In addition, mothers-to-be may also require other ophthalmic drugs, *e.g.* a course of antibiotic therapy may be required. Thomseth and associates (2015) published a nationwide cohort study on exposure to topical chloramphenicol during pregnancy (966 372 births 1997 - 2011, 6024 women exposed to topical chloramphenicol during the first trimester) to evaluate the risk of congenital malformations. The authors found no association with the use of chloramphenicol, in agreement with a previous study which analyzed the risk after systemic administration of the drug [48].

### 1.4 Elderly People

The elderly constitutes another group of at-risk patients. Elderly patients often suffer from a number of diseases and polypharmacy. In these subjects, the effects of ophthalmic medications on autonomic nervous system, as in the case of vasoconstrictors (tetrahydrozoline), mydriatics (phenylephrine, cyclopentolate, tropicamide), anti-glaucoma drugs (dipivefrine, brimonidine, apraclonidine), can induce unpleasant sympathomimetic adverse effects. Patients treated orally with adrenergic beta-receptor blocking drugs (“beta blockers”) are at- risk of adverse cardiac effects if ophthalmic timolol is dispensed. In some at-risk patients it is even recommended that ECG should be registered prior to the initiation of ophthalmic treatment with timolol or betaoxol [11]. Even in apparently healthy subjects, adrenergic beta-antagonists have induced marked bradycardia, even other arrhythmias and possibly also bronchoconstriction and hypotension, especially if administered in combination with other cardiovascular or psychoactive drugs. Other less frequent adverse problems are central effects such as anxiety, depression, confusion, hallucinations and sedation. Topically administered timolol eye drops are not the only ophthalmic preparations to cause severe confusion; scopolamine can also cause such episodes, especially in elderly people. Acetazolamide administered orally is another ophthalmic therapy capable of triggering mental confusion.

Huber and co-workers reported a material from 8 685 nursing home residents with almost 90 000 drug prescriptions (average 6.0 +/- 3.3 different drugs) [49]. Glaucoma was recorded in 520 (6.0%) of the residents. The authors concluded that anti-glaucoma pharmacotherapy was frequently prescribed in the context of polypharmacy. Similar observations with an even higher percentage (23%) were reported from Australia in a group of patients aged 75-84 years who were being treated with both topical and systemic beta-blockers [50]. In such a situation, the efficacy and safety of local and systemic therapies are modified and harmful drug-drug interactions become possible. It is not known whether ophthalmic beta-blockers can mask the symptoms of hypoglycemia induced by antidiabetic drugs, as is the case with oral beta-blocker administration.

### 1.5 Other Adverse Effects

Ophthalmic preparations can induce local effects in tissue situated close to the eyes, for example coloring of cheek or eyelids and growth of eyelashes (prostaglandin derivatives). In addition to local allergic reactions, ophthalmic agents can also cause generalized dermatological reactions including toxic epidermal necrolysis, anaphylaxis, fixed drug eruption, lichenoid drug reaction and systemic contact dermatitis [51].

### 1.6 Adverse Ocular Effects Caused by Systemic Drugs

Many systemic drugs given as either acute or chronic therapy may cause ophthalmic adverse effects. This includes drugs which directly influence the autonomic nervous systems, *i.e.* its adrenergic or cholinergic arms, or drugs which are indicated in other diseases but have autonomic side- effects, *e.g.* antipsychotic or antidepressant drugs which may antagonize the cholinergic system and thus increase intraocular pressure (IOP) especially when not in balance. This can also be seen during treatments with MAO-inhibitors, anti-Parkinson drugs, some antihistamines and cytotoxic drugs. It is well known that chronic glucocorticoid therapy can increase IOP and promote cataract development. Hydroxychloroquine and chloroquine, two drugs used for decades for the prevention of malaria and to treat rheumatic and other autoimmune diseases, are irreversibly toxic to the retina [52]. Furthermore, the toxic effects of ethambutol on the optic nerve may also be irreversible. The possibility has also been raised that nonsteroidal anti-inflammatory drugs may induce visual side- effects due to retinal toxicity [53]. Very occasionally bisphosphonate therapy has been associated with orbital inflammation [54]. Corneal epithelial changes can be induced by drugs in many pharmacological classes; they occur as an accumulation of phospholipids, vortex keratopathy or crystalline epithelial precipitation and blurred vision [55]. Toxic epidermal necrolysis and Stevens-Johnson Syndrome are both rare, acute, life-threatening dermatologic disorders involving the skin and mucous membranes and occasionally also the eye [56]. Recently three comprehensive reviews have been published on systemic drug therapy inducing ophthalmic adverse drug reactions [57-59].

## CONCLUSION

One must remember that local ocular therapy involves high concentrations of drug molecules in a small volume, *i.e.* one drop, and may cause marked, even dangerous systemic effects especially in newborns, small children and elderly patients. Aged patients often suffer from a number of diseases and may therefore be receiving systemic medicines, *i.e.* “polypharmacy”. This markedly increases the risk of harmful drug-drug interactions with ophthalmological preparations. The ophthalmologist should be aware of this possibility and carefully determine the patient’s drug history, including so-called “natural products”. Even though the data presented here is based on case reports, the physician should keep in mind that “my case might be one of the first”.

## Figures and Tables

**Table 1:** Ocular drugs. Reported systemic contraindications and systemic adverse effects of antiglaucoma medications. Contraindication involves hypersensitivity to ingredients or adjuvants. Reference: Finnish Pharmaca Fennica.

**ANTIGLAUCOMA MEDICATIONS**	**SYSTEMIC CONTRAINDICATIONS**	**REPORTED SYSTEMIC ADVERSE EFFECTS (USUALLY RARE)**
**Alpha-agonists**		
Apraclonidine	MAO inhibitors, children	Dry mouth, drowsiness, tachyarrhythmias, depressio, nausea, vasodilatation, taste disturbances, dyspnea, rhinorrea, constipation, dermatitis, skin rash
Brimonidine	Nursing mothers, infants, MAO inhibitors, tricyclic antidepressants, with caution in depression	Dry mouth, drowsiness, dizziness, tachyarrhythmias, headache, arterial hypo / hypertension, heart failure, taste disturbances, symptoms in upper airways, gastrointestinal-symptoms, skin rash
**Beta-blockers**		
Betaxolol (selective)	Asthma, emphysema, COPD, congestive heart failure, bradycardia, sick sinus syndrome, AV block (II,III)	Milder but similar systemic side- effects as timolol
Timolol (non- selective)	Asthma, emphysema, COPD, congestive heart failure, bradycardia, sick sinus syndrome, AV block (II,III)	Usually well tolerated, bradycardia, hypotensio, headache, dizziness, depression, hallucinations, anxiety, mental confusion, hair loss, broncho-obstruction, AV-block, urticaria, hypoglycemia, myastenia gravis, hair loss, anaphylaxis, insomnia, nausea, dry mouth, cough, lowered libido, muscle pain
**Carbon anhydrase inhibitors**	Sulfonamide allergy, metabolic acidosis, with caution in the presence of hypocalcemia, hyponatremia	
Acetazoleamide systemic	Renal acidosis, Addison's disease, severe renal/liver dysfunction	Paresthesia, malaise, fatigue, gastrointestinal disturbances, renal disorders, blood dyscrasias, metabolic acidosis, diarrhea/constipation, fever, urticaria, confusion, sleepiness, depression, gout, renal stones, taste disturbances
Brinzolamide	Severe renal insufficiency, hyperchloremic acidosis	Taste disturbances, nasopharyngeal infections, changes in red blood cell count and in blood chloride concentrations, depression, apathy, lowered libido, nightmares, insomnia, amnesia, dizziness, tinnitus, palpitations, angina pectoris, respiratory and asthma symptoms, epistaxis, sore throat, cough, gastrointestinal disturbances, eczema, muscle pain, malaise
Dorzolamide	Severe renal insufficiency, hyperchloremic acidosis	Headache, dizziness, paresthesias, epistaxis, malaise, dry mouth, renal stones, fatigue, eczema, itchiness, Stevens-Johnson syndrome
**Cholinergics**		
Pilocarpine	None	Headache, vertigo, malaise, asthma symptoms
**Prostaglandin analogues**		Skin pigmentation
Bimatoprost	None	Headache, tachycardia, hypertension, nausea, skin rash, dizziness, abnormal liver values
Latanoprost	None	Headache, tachycardia, skin rash, dizziness, exacerbated asthma symptoms, muscle and joint pain
Tafluprost	None	Headache, hypertension, exacerbated asthma symptoms, asthenia, abnormal liver values
Travoprost	None	Taste disturbances, dizziness, visual field defects, headache, tachy / bradycardia, hypo/ hypertension, asthma, difficulty in breathing, cough, dysphonia, sore throat, dermatitis, changes in hair structures, musculoscleletal pain, weakness, malaise

**Table 2:** Ocular drugs. Reported systemic contraindications and systemic adverse effects of other ophthalmic drugs. Contraindication involves hypersensitivity to ingredients or adjuvants. Reference: Finnish Pharmaca Fennica.

**ANTIHISTAMINICS**	**SYSTEMIC CONTRAINDICATIONS**	**REPORTED SYSTEMIC ADVERSE EFFECTS (USUALLY RARE)**
Azelastine	None	Eczema, itching, disturbance to taste
Emedastine	None	Headache, disturbance to taste, eczema, weird dreams
Ketotifen	None	Headache, eczema, dry mouth, drowsiness
Levocabastine	None	Angioneurotic edema, urticaria, headache
Olopatadine	None	Rhinitis, headache, dysgeusia, eczema, fatigue
**ANTIMICROBIALS**		
Aciclovir	None	None
Chloramphenicol	None	Aplastic anemia, agranulosytosis, peripheric neuropathy
Fucidinic acid	None	Eczema, itching
Levofloxasin	None	Eczema, headache, rhinitis
Ofloxacin	None	Eczema, headache, rhinitis
Tobramycin	None	Headache, laryngospasm
**INHIBITORS OF HISTAMINE RELEASE**		
Lodoxamide	None	Headache, vertigo, fatigue, malaise, eczema, rhinitis
Cromoglycate	None	None
**LOCAL ANESTHETICS**		
Oxybuprocaine	None	Bradycardia, hypotensio, vertigo, tremor
**MOISTENING PREPARATIONS**	None	None
**MYDRIATICS**		
Atropine	None	Dry mouth, skin redness, tachycardia, gastrointestinal symptoms
Cyclopentolate	None	Fatigue, confusion, anxiety, hallucinations
Phenylephrine	None	Hypertension, reflectory bradycardia, tachycardia, palpitations, headache, tremor, sweating
Scopolamine	None	Dry mouth, constipation, confusion, anxiety, drowsiness, hallucinations
Tropicamide	None	Dry mouth, constipation, confusion, anxiety, drowsiness, hallucinations
**NON-STEROIDAL ANTI-INFLAMMATORY DRUGS**		
Bromfenac	Acetylsalicylic acid (ASA) sensitivity	Epistaxis, cough, rhinitis, asthma symptoms, face swelling
Diclofenac	Acetylsalicylic acid (ASA) sensitivity	Itching, cough, rhinitis
Ketorolac	Acetylsalicylic acid (ASA) sensitivity	Headache, bronchospasm, asthma symptoms
Nepafenac	Acetylsalicylic acid (ASA) sensitivity	Headache, malaise, cutis laxa
**STEROIDS**		
Dexamethasone	None	None
Fluorometholone	None	Hypercorticoidism
Prednisolone	None	Headache, itching, tastedisturbances
**VASOCONSTRICTORS**		
Tetrahydrozoline	None	Hypertensio, palpitatio, sweating, anxiety, fatigue
**VEGF INHIBITORS**		
Aflibersept	None	Theoretical risk of arterial thromboembolic attacks
Ranibizumab	None	Headache, nasopharyngitis, joint pain, anemia, anxiety, cough, malaise, theoretical risk of arterial thromboembolic attacks
